# Do Domestic Dogs Understand Human Actions as Goal-Directed?

**DOI:** 10.1371/journal.pone.0106530

**Published:** 2014-09-17

**Authors:** Sarah Marshall-Pescini, Maria Ceretta, Emanuela Prato-Previde

**Affiliations:** 1 Sez. di Neuroscienze, Dipartimento di Fisiopatologia medico-chirurgica e dei Trapianti, Università di Milano, Milan, Italy; 2 Comparative Cognition, Messerli Research Institute, University of Veterinary Medicine, Vienna, Medical University of Vienna, University of Vienna, Vienna, Austria; 3 Wolf Science Centre, Ernstbrunn, Austria; University of Portsmouth, United Kingdom

## Abstract

Understanding of other’s actions as goal-directed is considered a fundamental ability underlying cognitive and social development in human infants. A number of studies using the habituation-dishabituation paradigm have shown that the ability to discern intentional relations, in terms of goal-directedness of an action towards an object, appears around 5 months of age. The question of whether non-human species can perceive other’s actions as goal-directed has been more controversial, however there is mounting evidence that at least some primates species do. Recently domestic dogs have been shown to be particularly sensitive to human communicative cues and more so in cooperative and intentional contexts. Furthermore, they have been shown to imitate selectively. Taken together these results suggest that dogs may perceive others' actions as goal-directed, however no study has investigated this issue directly. In the current study, adopting an infant habituation-dishabituation paradigm, we investigated whether dogs attribute intentions to an animate (a human) but not an inanimate (a black box) agent interacting with an object. Following an habituation phase in which the agent interacted always with one of two objects, two sets of 3 trials were presented: new side trials (in which the agent interacted with the same object as in the habituation trial but placed in a novel location) and new goal trials (in which the agent interacted with the other object placed in the old location). Dogs showed a similar pattern of response to that shown in infants, looking longer in the new goal than new side trials when they saw the human agent interact with the object. No such difference emerging with the inanimate agent (the black box). Results provide the first evidence that a non-primate species can perceive another individual’s actions as goal-directed. We discuss results in terms of the prevailing mentalisitic and non-mentalistic hypotheses regarding goal-attribution.

## Introduction

Intention attribution is considered a fundamental ability underlying much of cognitive, social and linguistic development in human infants and much debate has revolved around when infants start viewing other’s actions as intentional [1], [2]. The emergence of a fully-fledged mentalistic understanding in children appears to occur around the age of 4 years, with the understanding of false-belief [3], [4] however, understanding of actions as goal-directed has been considered a necessary precondition for understanding intentional actions (and attributing mental states) and a number of studies have shown the emergence of this ability before the end of the first year [5], [6], [7].

Indeed in a seminal work using an habituation-dishabituation paradigm, Woodward found that when 5-month old infants repeatedly observe a person interacting with an object, they will then look longer when the actor suddenly switches to interacting with a different item in the same location than when they see the actor interacting with the usual object placed in a new location. The ‘surprise’ shown by infants in this paradigm has lead authors to conclude that infants understand the actor’s action as being goal-directed to a particular target [7], [8]. Further studies using a similar paradigm have shown that infants attribute goal-directedness to animate but not inanimate agents [7], [9] (although inanimate agents can also ‘have goals’ if they exhibit certain features [10], [11]) and to deliberate but not accidental actions towards an object [12], [13].

Overall, these findings suggest that infants represent actions as organized by the relation between agent and object: however, there is currently a lively debate around the question as to whether infant’s ability to understand such a relation is based on their interpretation of the mental connections between the agent and the object (e.g. the actor ‘wants/prefers’ that particular target) [13] or whether results can be equally thoroughly explained by a more functional interpretation where goals are defined as the perceivable outcomes (or targets) of specific actions and inferential reasoning about the relationship between the elements is sufficient to allow infants to ‘solve’ such apparently mentalistic questions [14].

Attribution of intentions in non-human animals has been even more controversial to establish [15], but gathering evidence in the past 15 years suggests that non-human primates may discern goal-directed actions in certain contexts. For example, both chimpanzees and capuchin monkeys distinguish between a person who is unable vs. unwilling to give out food, and the latter have been shown to do so only with animate but not inanimate agents [16], [17]. Using a looking time paradigm similar to Woodward’s method with infants, it was found that rhesus macaques expect people to reach towards an object they have been oriented to, suggesting that they use head orientation to predict action [18] and marmosets attribute goal-directed action to conspecifics and humans, less to a marmoset-like robot, and not at all to an inanimate black box [19], [20]. Furthermore, as with human infants [21], [22] attribution of goal-directed behaviour in macaques may be mediated by one's own experience of the world, since familiar (but not unfamiliar) actions observed being performed by a human are interpreted as goal-directed [23]. Finally, chimpanzees, similarly to infants [24] have been found to complete unfulfilled goals, rather than reproduce what the experimenter was attempting (but failing) to achieve [25], [26] and imitate rationally—that is, they skip certain actions that the experimenter produced due to her physical constraints [27].

In the current study, we adapted the method used by Woodward and colleagues [7], [9] to start investigating the perception of (human) goal-directed action in dogs. Dogs have been considered by a number of researchers as a potentially interesting case because of their approximately 30.000 years of association with humans [28], [29], which may have resulted, through a process of ‘convergent evolution’, in more ‘human-like’ socio-cognitive skills in dogs [30]–[33].

As regards dogs’ potential understanding of human intentions, research so far shows that dogs can distinguish friendly vs. threatening behaviours enacted by both familiar and unfamiliar humans, and respond differently depending on whether they are exhibited in a playful or ‘serious’ context [34]. Furthermore, they follow pointing gestures more in collaborative than competitive situations [35] and appear to appreciate the referential nature of the pointing gesture [36], [37]. Moreover, in a recent study dogs were shown to preferentially follow intentional pointing accompanied by ostensive communicative cues (e.g. gaze alternation) compared to morphologically similar but unintentional ones (e.g. pointing whilst alternating gaze from wall to wrist watch) [38]. Hence, it seems that dogs may be viewing human pointing as a goal-directed act, although no study has directly addressed this issue and results contrast with an earlier study in which it was shown that although dogs can use a marker to locate food in an object-choice task, they do not differentiate between an intentional vs. accidental placing of the marker on the object [39]. Finally, similarly to studies with infants, dogs have been shown to imitate selectively in that they will reproduce specific actions taking into account the constraints of the model, suggesting they are sensitive to the efficiency of goal-directed actions [40] but see also [41], [42]. Taken together results from these studies are inconclusive, although some at least seem to suggest that dogs may recognize when a human or a conspecific’s action directed towards an object is goal directed.

Interestingly, most of the studies investigating dogs’ understanding of an actor’s intentions have been set in a communicative context (e.g., object-choice tasks in which an actor shows the dog where the food is hidden) [38], [39], whereas this has not been the case for neither infants nor primates [43]. Hence in the current study we adopted Woodward’s and colleagues looking time habituation-dishabituation paradigm to investigate dogs' understanding of human object-directed actions, with no communicative cues being displayed towards the subject. The overall aims of the study were to assess whether: i) Woodward’s habituation-dishabituation experimental paradigm could be used with dogs and whether ii) results with dogs could be comparable to those with infants, in showing attribution of goal-directed actions to an animate (a human) but not an inanimate (a black box) agent interacting with an object.

Hence, we presented dogs with either a black box or an unfamiliar individual interacting repeatedly with one of two distinct objects (a globe vs. a watering-can). As in paradigms with infants, once dogs had habituated to the scene (habituation phase), the position of the objects was switched (test phase). Dogs were then given three trials in which they saw the agent (animate or inanimate) interact with the usual object (in the new location), followed by three trials in which the agent interacted with the new object (in the familiar location) in a counterbalanced order across subjects. In infant studies, following the habituation phase, results showed that whereas there was a significantly higher looking time in the new object compared to the new location trials when an animate agent (i.e. a human hand) grasped for the object, no such effect was discernable when an inanimate agent (e.g. mechanical claws, rods etc.) carried out the same behaviour [7], [9].

Similarly if dogs, perceive the actions of the animate agent as goal-directed, we expect a significantly higher looking time when they see the person (animate agent) interact with the novel object compared to the usual one (in the new place) but this pattern of results should not however occur with the inanimate object (i.e. the black box).

## Method

### Ethics statement

No special permission is required for use of animals (dogs) in such behavioural studies in Italy. The relevant ethical committee is the Ethical Committee of the Università degli Studi di Milano. Dog owners were briefed as to what would happen during the test and gave consent before the test could commence.

### Subjects

Fifty-two dog-owner dyads were recruited based on the *Canis sapiens Lab* (University of Milan) database. The dog sample consisted of 22 males and 30 females whose ages ranged from 1 to 10 years (mean = 5.9 years, SD = 2.6). 31 subjects were pure-breed and 21 mixed-breed (see Appendix 1 for breed of dogs). All the dogs were kept for companionship, lived within the human household and had either no or only basic training experience. Most dogs had participated in other studies by our group, but not studies using the experimental paradigm adopted here. Dogs were randomly assigned to one of two groups: Animate, Inanimate.

### Material

Two objects (a watering can and a globe) were used, following a preliminary test in which a different sample of 7 dogs showed no preference for either of them, neither in terms of first choice (binomial p = 0.4) nor interaction time (t = 2, p = 0.09).

#### Procedure

Dogs were briefly allowed to explore the testing room, whilst the owner was briefed regarding procedure. Then the owner sat in a chair, positioned 30 cm behind the dog. The dog wore a short leash (60 cm) attached to their collar/harness, and the leash was fixed to a hook in the wall. Owners were asked not to interact with the dogs throughout the test, and to allow them to sit or lie down as they wished. A video camera was placed directly in front of the dog (2 m away) so as to capture the dog’s looking behaviour (but not the agent’s actions). On either side (at equal distances) of the videocamera two objects, a globe and a watering can, were placed on the ground 110 cm apart. A door (from which the experimenter entered during trials) was located 120 cm behind the videocamera.

Two people were involved in testing: the experimenter and the observer. The experimenter acted out the required scene (in the Animate group) and controlled the black box from behind the curtain (in the Inanimate group). The observer coded the dog’s behaviour from a computer located in an adjacent room and linked to the videocamera in the testing room.

Prior to the start of each trial the experimenter briefly knocked on the door to attract the dog’s attention (if dogs habituated to this and stopped responding, a hand-clap was used as an alternative).

#### Habituation Phase

In the habituation phase dogs in the Animate group then saw the experimenter (a female) entering the room, approaching, crouching down and interacting with one of the objects by touching it repeatedly and looking at it intently. The duration of the trial (i.e. the time the experimenter spent interacting with the object) was dependent on the dog’s looking behaviour, in that it lasted until dogs looked away for more than 2 seconds or for a maximum trial length of 12 seconds. On hearing the predetermined signal from the observer (see below), the experimenter got up and without looking at the dog left the room. After a brief pause (5 seconds) the experimenter knocked on the door again to attract the dog’s attention and start the subsequent trial.

In the Inanimate group the door was left open, but a curtain was placed in front of it. A black box (w 18 cm, h 37 cm, d 23 cm) was placed centrally, in front of the curtain, and was manipulated by the experimenter (sitting behind the curtain) using a long (68 cm) stick attached to it. During the habituation phase, after drawing the dog’s attention by knocking on a board behind the curtain, the experimenter pushed the black box towards one of the objects and continued moving it so that it repeatedly bumped into the object, from different angles, following the same trajectory used by the animate agent. Dogs could see neither the experimenter nor her hands manipulating the stick. The duration of the trial was dependent on the dog’s looking behaviour, and lasted until dogs looked away for more than 2 seconds or for a maximum of 12 seconds. On hearing the predetermined signal, the experimenter pulled the box back to the central location in front of the curtain and after a brief pause (5 seconds), knocked again to start the subsequent trial.

The observer coded the dogs’ looking behaviour (for both groups) by watching the dog on a computer monitor (in the adjacent room) linked to the video-camera in the testing room. The coder pressed a key on a computer keyboard when the dog looked towards the object the experimenter was interacting with. A computer program calculated looking times and habituation criteria from this input (Habit 1.0, University of Texas). The observer was unaware of the order of test trials assigned to the dog (i.e. whether it was new side followed by new goal or viceversa), however she was aware of group (Animate vs. Inanimate) and when the habituation ended and the test trials began. Unfortunately, the observer reported it was easy to ‘guess’ where the experimenter was located in the room by the dogs’ looking behaviour, hence ‘live’ coding could not be considered blind (see later ‘analyses’ section for coder-reliability to counter this problem).

The dogs’ looking was timed starting when the actor’s hand (or the black box) made contact with the object and continuing until the dog had looked away from the person/box for 2 s or until 12 s had elapsed. Thus, looking was timed as the dog saw the agent interacting with the objects. To let the observer know when to start coding the experimenter signalled by emitting a ‘beep’ from a stopwatch when she (or the black box) started touching the object. Similarly, the observer activated the stopwatch with a beeping sound to signal to the experimenter when to end the test (i.e. after the dogs had looked away for more than 2 s or after 12 s).

For half of the dogs, the object on the right was the target of the agent’s interaction, for the other half the object on the left was. Since side placement of the globe and watering-can was counterbalanced, there were four possible habituation events for each group. At least 6 dogs in each group were habituated to each of the four events. Males and females were distributed approximately evenly across habituation events.

The habituation criterion was computed for each dog. A dog reached habituation criterion after three consecutive trials in which the duration of its looking time behaviour towards the event totalled to less than half of the sum of the three consecutive trials with the longest exhibited looking time (e.g. if the longest duration of looking time exhibited by the dog in three consecutive trials were respectively 10, 9, 11 seconds, habituation would be reached by this dog when the sum of the looking time duration in three consecutive trials occurring after the above, was less than 15 seconds i.e. (10+9+11)/2). Hence each dog had a minimum of six trials in the habituation phase (if the sum of the looking duration in the last three consecutive trials was less than half the sum of the looking duration in the first three trials). Once a subject had reached the habituation criterion an extra habituation trial was presented to obtain a measure of the dog’s looking time following habituation. A predetermined maximum of 14 habituation trials was set, after which dogs were presented with test trials whether they had habituated or not [7]. The length and number of habituation trials presented was therefore subject-determined, although a maximum number of trials was set (see [44] for the necessity of subject-determined habituation and a discussion of methodology).

#### Test Phase

**Figure 1:**
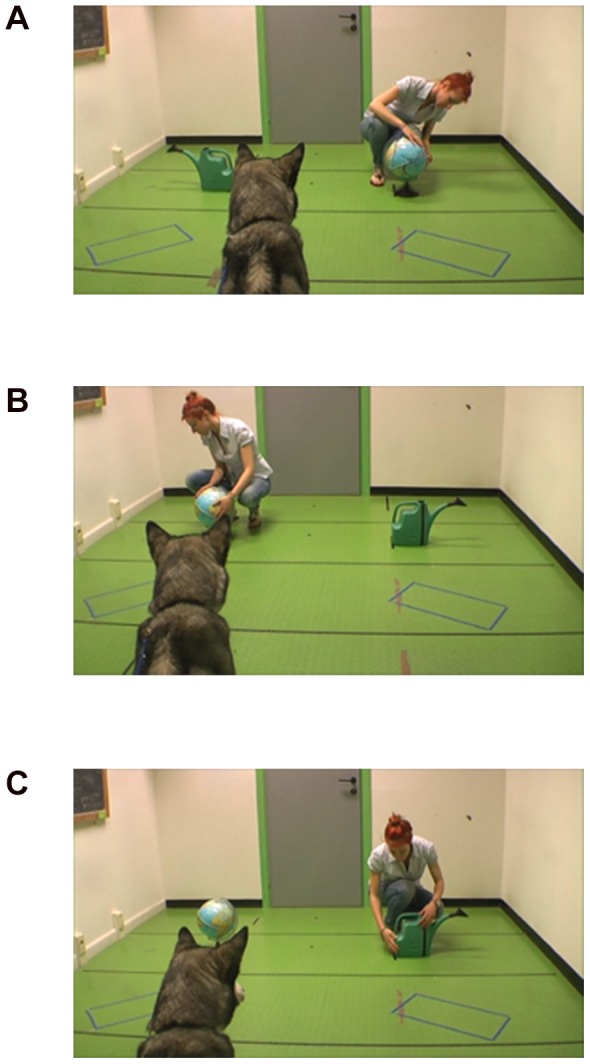
Photographic representation of the experimental setup. A) In the habituation phase dogs in the animate group saw the experimenter interacting repeatedly with one of two objects (in this case the globe). After a subject-determined number of trials to reach habituation, the dog was presented with B) 3 new-side trials (the experimenter interacted with the same object placed in the new location) followed by C) 3 repetitions of the new-goal trial (the experimenter interacted with the novel object, the watering-can here, placed in the old location).

After the habituation trials, the owner was asked to leave the room with the dog for approximately 1 minute. During this exit the researcher swapped the position of the objects. The dog and owner re-entered the room and took up their usual position. Following the same procedure used with infants [7] the dog was then given one familiarization trial (lasting 12 seconds max or after the dog looked away for more than 2 seconds, as above) with the objects in their new positions but no actor present, so that any increased looking time in test trials could not be due simply to the new disposition of the objects. The dog then saw three consecutive new-goal test trials followed by three consecutive new-side test trials. On new-goal events the agent approached the same side as during habituation trials, but this time interacting with the other object. On new-side events the actor approached and interacted with the same object as in habituation trials, which was now placed on the other side of the room (Figure 1). The order of the test events (three new-goal followed by three new-side first) was counterbalanced across dogs in all groups.

### Analyses

Since the observer during ‘live’ coding reported that it was in most cases easy to guess the order of test trials by the dog’s direction of looking behaviour, 4 months after the test was conducted, the same observer re-coded all trials (habituation and test phase) a second time from video (using the Habit program). However, to obtain a coding that was blind to test order trials, this time the observer coded the test trials first (hence without knowing what the prior habituation event had been) and the habituation trials last. Intra-observer reliability on the entire dataset between ‘live’ coding and second coding was found to be excellent (Cronbach’s alpha  = 0.88). However, a few discrepancies occurred with three dogs who had seemingly habituated after fewer than 14 trials during live coding, who were in fact found not to have done so on second coding (they were hence eliminated from the subject pool for analyses, see below). Data from the second coding was used for statistical analyses since it was thought to be more accurate, having been collected without at the same time the necessity of communicating with the actor during trials and with less of a potential bias due to ‘guessing’ the test order. Furthermore, a second observer (SMP), completely blind to trial order and habituation condition, coded 50% of test trials randomly selected across groups. Inter-observer reliability done between the second coding of MC and SMP was good (Cronbach’s alpha = 0.82). Finally, an observer unrelated to the study, who had no knowledge of the hypothesis being tested coded the dogs’ looking behaviour in a semi-randomly chosen selection of habituation and test trials presented non-sequentially (i.e. habituation and test trials were interspersed). This naïve observer coded 20% of the data (half from the animate agent half from the inanimate agent group) and the reliability between her scoring and MC’s second scoring (used for analyses) was also high (Cronbach’s alpha = 0.8).

A Chi-square test was used to compare groups on the number of dogs that did not habituate. The mean looking time across the first three and last three habituation-trials as well as across the new goal and new side trials, was computed for each dog. A first Generalized Estimated Equation (GEE) with Bonferroni corrected posthoc comparisons was run with group (Animate vs. Inanimate), test condition (new goal vs. new side trials) and test order (new goal – new side vs. new side new goal) as independent variables and mean looking time in each condition as dependent variable. Once established that test order had no effect on on looking time in test trials, we ran a full model testing our predictions and including sex and object (globe vs. watering can) used during habituation as potential confounding variables.

Hence a GEE (with Bonferroni corrected posthoc comparisons) was run, with group (Animate vs. Inanimate), condition (first habituation three, last habituation three, new goal, new side trials), habituation object and sex as independent variables and mean looking time across the three trials in each condition as dependent variable. A final GEE (with Bonferroni corrected posthoc comparisons) was run, with group (Animate vs. Inanimate) and condition (habituation vs. new goal vs. new side trials) as independent variables, but in this case the dogs’ looking time in the extra habituation trial, first new goal and first new side test trials was used.

## Results

Four dogs did not complete the test because they became distressed hiding under the owner’s chair becoming invisible to the coder (1) or for procedural errors (3). A further dog was excluded because the combination of black eyes and dark shaggy hair made it impossible to discern her looking behaviour. Three dogs after the second coding were found not to have habituated in the number of habituation trials presented to them and hence had to be removed.

The proportion of dogs that did not habituate and the number of trials to habituation (for those who did habituate) did not differ between groups (N. of dogs that did not habituate: 4/23 in the Animate, none in the Inanimate *Χ^2^* = 4, p = 0.07; Median number of habituation trials for both groups was 9). The four dogs that did not reach habituation were excluded from further analyses hence the remaining group composition was as follows: 19 (13F, 6M; mean age: 5.6, range: 1.5–10) in the Animate, 21 (10F, 11M; mean age: 6.1, range: 1–11) in the Inanimate group. However, it is important to note that results did not change when including the four dogs that did not reach habituation.

The GEE assessing the potential effect of presentation order (i.e. new side - new goal vs. new goal - new side) on subject’s looking time in test trials, showed no main effect of presentation order (Wald = 1.8, df = 1, p = 0.2) and no interaction between presentation order and neither condition (Wald = 0.9, df = 2, p = 0.6) nor group (Wald = 5, df = 2, p = 0.075).

The GEE with the mean looking time across the first three and last three habituation trials, the new side and new goal trials as dependent variable, showed a significant group*condition interaction (Wald = 18.9, df = 3, p<0.001) and a main effect of condition (205.2, df = 3, p<0.001). There was however no main effect of group (Wald = 0.09, df = 1, p = 0.8), sex (Wald = 1.2, df = 1, p = 0.3), or habituation object (Wald = 0.008, df = 1, p = 0.9).

There was no difference between groups in the time spent looking at the scene in neither the first nor the last three habituation-trials (mean first 3 habituation trials: Animate = 7, Inanimate = 7.8, p = 1; mean last 3 habituation trials: Animate = 2.7, Inanimate = 3.1; p = 1).

In the Animate group, dogs spent significantly less time looking at the scene in the last three compared to the first three habituation trials (mean: first 3 habituation trials = 7, last 3 habituation trials = 2.7 p<0.001). Furthermore, dogs in this group recovered from habituation (last three trials) in new goal trials (mean: habituation = 2.7, new goal = 6.5, p<0.001) but not in new side trials (mean: habituation = 2.7, new side = 4, p = 0.6). Finally, dogs in this group looked at the interaction for significantly longer in new-goal compared to new-side trials (mean: new-side = 3.9 vs. new-goal = 6.5, p = 0.02).

**Figure 2:**
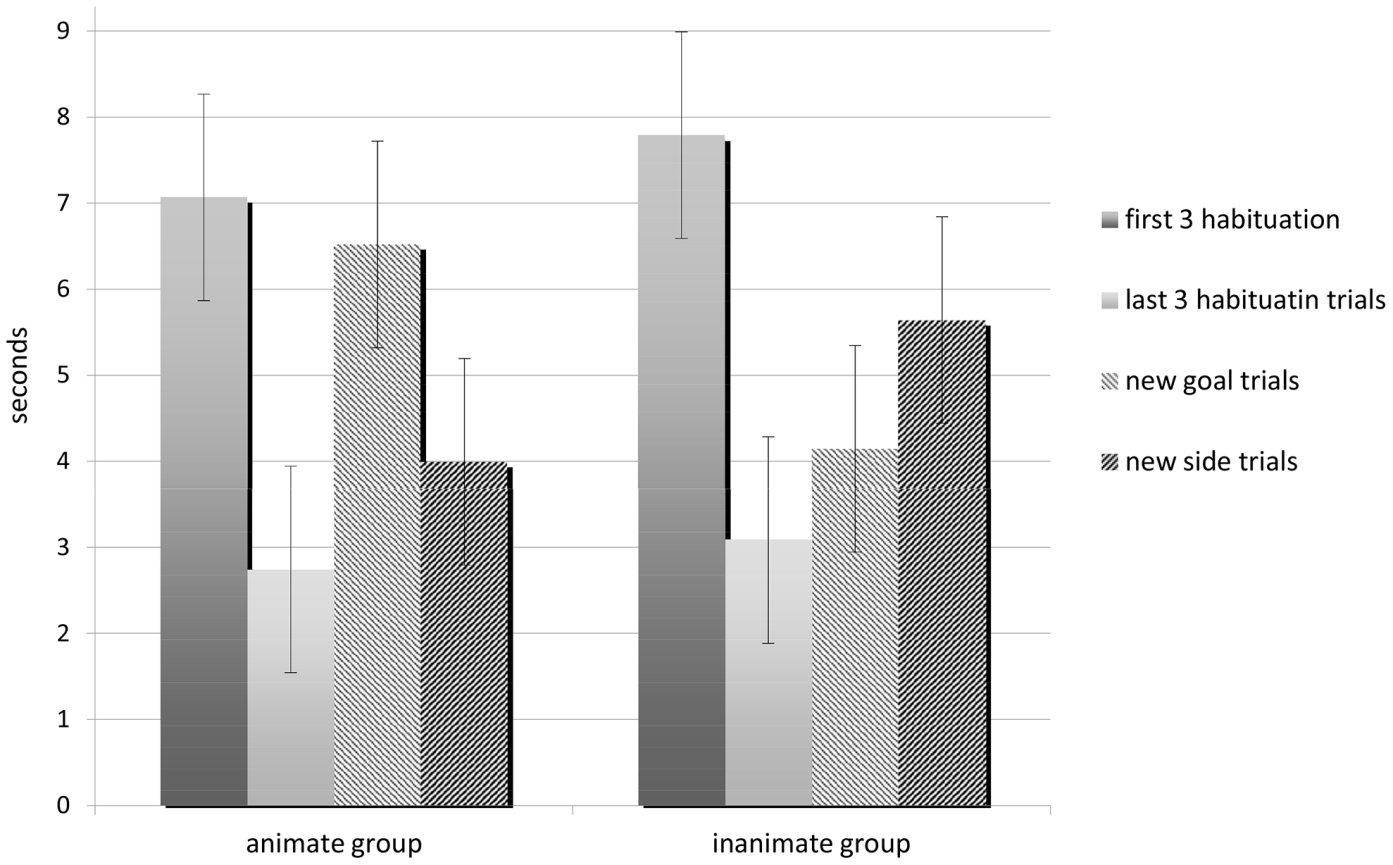
Dogs’ looking time. Mean looking time (and 95% confidence interval) across the first and last three habituation trials, new-side trials and new-goal trials for dogs in the animate and inanimate group.

In the Inanimate group, dogs also spent significantly less time looking at the scene in the last three compared to the first three habituation trials (mean: first 3 habituation trials =  7.8, last 3 habituation trials = 3.1 p<0.001). However, a reverse pattern emerged with dogs recovering from habituation in new side trials (mean: habituation = 3.1, new side = 5.6, p<0.01) but not in new goal trials (mean: habituation = 3.1, new goal = 4.1, p = 1). Furthermore, there was no significant difference in dogs’ looking time in new side vs. new goal trials in this group (mean: new-side = 5.6 vs. new-goal = 4.1, p = 0.19) (Figure 2).

The GEE with looking time in the extra habituation trial, 1^st^ new side and 1^st^ new goal trial as dependant variable also revealed a group*condition interaction (Wald = 8.9, df = 2, p = 0.01). There was no difference between groups in the time spent looking at the scene in the extra habituation trial (mean: animate = 2.6, inanimate = 5.9; p = 0.2). In the animate group, dogs recovered from habituation in the 1^st^ new goal trial (mean: extra habituation = 2.5, 1^st^ new goal = 6.9, p = 0.009) but not in the 1^st^ new side trial (mean: extra habituation = 2.5, 1^st^ new side = 5.9, p = 0.2). There was however no difference between the 1^st^ new goal and 1^st^ new side trial (mean: new-side = 5.9 vs. new-goal = 6.9, p = 1). In the Inanimate group there was no recovery from the extra habituation trial neither in the 1^st^ new goal nor 1^st^ new side trial (mean: extra habituation = 5.9, 1^st^ new side =  7, p = 1; 1^st^ new goal = 4.8, p = 1), nor was there a difference between the latter test trials (P = 0.3).

As for the dogs’ choice of the object when they were allowed to freely explore the room at the end of the test: 3 of the 19 dogs in the Animate group approached the new goal object first, 2 the new side, and 12 did not move from their owner’s side. Of the remaining two dogs the object choice was lost due to video malfunction. In the Inanimate group 4 of the 21 dogs in the animate group approached the new goal object first, 3 the new side, and 13 did not move from their owner’s side, and the remaining dog went to explore the curtain before either of the objects. Hence overall there was no difference in the approach behaviour of dogs in the different conditions.

## Discussion

Dogs readily habituated to the repeated presentation of the actor/box interacting with one of the objects. Furthermore, the habituation rate, proportion of dogs habituating and duration of looking time in the latter three habituation trials was comparable across groups, and comparable to results from the infant literature (e.g. the average looking time in the last 3 habituation trials for babies was approx. 3.1 seconds [7] and for dogs 2.9). Interestingly, dogs showed a similar pattern of results to human infants and marmosets, in that they looked significantly longer at the new goal trials compared to the new side trials when the actor was an animate agent but not when the agent-object interaction was performed by an inanimate agent [7], [9], [19], [20].

Indeed it seems that dogs, when seeing the inanimate agent touch the objects, focused on the path taken by the black box, as suggested by dogs’ recovery from habituation in in new-side trials (when the path had changed) but not in new-goal trials. However, when dogs saw a person interact with the object they showed a tendency to look longer to a change in the object being manipulated (new goal trials) than to a change in the path taken by the actor (new side trials), suggesting that the dogs’ attention in this case was on the actors’ goals, i.e. the object they interacted with.

The first trial data comparing new-side to new-goal conditions was not as clear-cut, since indeed no difference emerged between conditions in the animate group, although recovery from habituation did occur in the first new-goal (but not new side) trial. One important aspect to note, is that the first trial of each condition is to a certain extent novel, hence it is likely to increase the subject’s looking time. In infant studies first trial data is not reported [7], [9], hence it is not easy to directly compare results from this study with the pattern of looking in the infant literature. Overall, whereas dogs’ looking time appeared comparable to that of infants in the habituation phase, in test trials it was overall shorter. Whether this is due to a lack of interest, or a species typical pattern of looking is difficult to establish without studies directly comparing the two species on multiple tasks.

Hence based on current results, and their similarity to the pattern of results obtained in similar infants studies, it appears that dogs interpreted the actor’s behavior towards the object as goal-directed.

However, dogs have been shown to be neophilic, in that given a choice between a familiar and a novel object they will prefer to interact with the latter [45]; furthermore, in a Visual Paired Comparison paradigm where old and new object are simultaneously presented on a screen, dogs looked preferentially at the novel projected object [46]. Interestingly, novelty in the current paradigm is designed to be present in both sets of test trials, the new side and new goal trials. Indeed novelty was perceived differently for the animate and inanimate agents: in fact dogs looked longer at the person when she suddenly interacted with a different object but did not show the same pattern of looking behavior when the black box changed objects. This suggests that dogs formed an expectation about the object directed goals of the human, but not of the inanimate object. Results with the human as the actor are further confirmed by a clear recovery in looking time from the last three habituation trials to the new goal but not the new-side trials, where an opposite pattern emerged with the inanimate agent, where dog’s looking time recovered in new-side but not new-goal trials. It is not altogether clear why dogs’ looking time increased significantly in new-side trials when the inanimate object was the actor; one possibility is that dogs formed an expectation of the black box’s trajectory based on previous trials, and hence showed a ‘surprise’ effect when the trajectory was suddenly different. However, further testing will be required to ascertain dogs’ expectations as regards inanimate agents.

One possible explanation for dogs’ increased looking time in new-goal trials with the human agent, is that based on test trials the dogs stopped expecting the experimenter to do something interesting and relevant to them with the old object, but based on previous experience with humans, they may still have expected something interesting for them to occur (e.g. playing with it or fetching a piece of food from it) when a new object was being manipulated. This possibility cannot be excluded, however it is perhaps interesting to note, that once the dogs were released very few investigated the objects at all, suggesting that there was no strong expectation of finding food. Furthermore, although the dogs may have had expectations of what humans usually do when interacting with an object in relation to themselves, in most cases before something relevant to them occurs, communicative cues such as using the dogs’ name or looking directly at it, are used to engage the dogs’ attention. Indeed such cues have been shown to be powerful attention getters in many studies [47], [48]. However in the current study no such cues were used, hence the actions may have been more equated to a person’s interacting with everyday object, actions largely irrelevant to dogs.

Representation of another’s actions as goal-directed has been shown in a number of primate species [16]–[18], [23], [49], but this is the first evidence, to our knowledge, of a similar phenomenon being shown in a non-primate species. However, numerous studies have shown dogs' sensitivity to their human partner’s attentional states, both in begging, play and communicative situations [50]–[54], and growing evidence, using diverse experimental paradigms, suggests that dogs have some basic understanding of human visual perspective taking [55]–[57]. Finally, dogs have been shown to imitate selectively, taking into account the contextual constraints of the demonstrator [40] but see [41], [42], which would also suggest some understanding of their conspecific demonstrator’s goals.

Current results add to the evidence suggesting that dogs may also have the ability to perceive human object-directed action as goal-directed. However, this does not necessarily imply a mentalistic understanding on the dog’s part. Indeed a number of authors have suggested that an understanding of goal-directed actions, even in infants, does not necessarily imply an understanding of mental states as representational. Some authors suggest that infants at this stage may have a perception of simple internal states but only referring to actual objects, rather than their representation [58], [59]; whereas other authors dismiss the looking time studies arguing that such data only provide evidence for infants abilities to form statistical associations during the course of an experiment [60]. A third non-mentalisitic approach suggests that prior to the emergence of a mentalistic interpretation of the agent action relationship, infants may adopt a teleological stance whereby through a process of inferential reasoning (the ‘rationality principle’) as regards the actions (and their efficacy), the goal states and the constraints in specific situations, an infant may be capable of inferring the latter’s goals without appealing to ‘desires or beliefs’ [2], [61].

At present it is premature to reach conclusions as to the underlying mechanisms driving dogs’ apparent perception of human actions as goal-directed. Considering dogs have been shown to apply ‘inferential reasoning’ in simple contexts [62], [63], and show sensitivity to situational constraints in a social learning context [40]–[42], the rationality principle may be sufficient to account for dogs’ attribution of goal-directed action to humans, at this point in time. However, differently from studies carried out with infants [5], [64], [65], a systematic investigation of this issue has not as yet been carried out and would be necessary to ascertain the validity of such an explanation. What is however important in the current context is that the ‘Woodward paradigm’ has been shown to be applicable to dogs, which opens up the possibility of further investigating the underlying mechanisms of their understanding of human actions as goal-directed, as well as how dogs perceive gazing and pointing (as has been done with infants see [66], [67]).

In conclusion, the current study suggests that a well-known and accepted method of investigation used in the study of infants’ understanding of others’ goal-directed action can be successfully employed with dogs. Dogs show a similar pattern of response to that shown in infants and marmosets, with a clear recovery in looking time when the animate (person) but not inanimate (black box) agent unexpectedly shifts their action to a novel object. This suggests that dogs may view the actions of humans (but not of black boxes) as goal-directed, although further studies are needed to clarify whether this process is based on mentalistic processes or inferential reasoning about the contingency of the situation.

## Supporting Information

Appendix S1 Breed of dogs participating in the study.
(DOC)
