# Supplementary material for: Do Domestic Dogs Understand Human Actions as Goal-Directed?
Source: PLoS One. 2014 Sep 17;9(9):e106530. doi: 10.1371/journal.pone.0106530 (PMC4167693; doi:10.1371/journal.pone.0106530)
Supplement: Appendix S1 — Breed of dogs participating in the study. (DOC) [file pone.0106530.s001.doc]

**Supporting Information**

**Text S1**

Breed of dogs participating in the study: 3 Labrador, 7 Golden retriever, Rhodesian ridgeback, 2 Border collie, Beagle, Irish setter, 2 Brittany spaniel, Flatcoated retriever, Doberman, Jack Russell terrier, Basenji, 3 German shepherd, Poodle, German hound, Cau de agua, Bolognese, Westhighland terrier, American Staffordshire, Sheltie.
